# Melarsoprol Cyclodextrin Inclusion Complexes as Promising Oral Candidates for the Treatment of Human African Trypanosomiasis

**DOI:** 10.1371/journal.pntd.0001308

**Published:** 2011-09-06

**Authors:** Jean Rodgers, Amy Jones, Stéphane Gibaud, Barbara Bradley, Christopher McCabe, Michael P. Barrett, George Gettinby, Peter G. E. Kennedy

**Affiliations:** 1 Institute of Infection, Immunity and Inflammation, College of Medical, Veterinary and Life Sciences, University of Glasgow, Glasgow, United Kingdom; 2 Laboratoire de Pharmacie Clinique, Nancy Université, Nancy, France; 3 Institute of Neuroscience and Psychology, Glasgow Experimental MRI Centre, University of Glasgow, Glasgow, United Kingdom; 4 Wellcome Trust Centre of Molecular Parasitology, Institute of Infection, Immunity and Inflammation, College of Medical, Veterinary and Life Sciences, University of Glasgow, Glasgow, United Kingdom; 5 Department of Mathematics and Statistics, University of Strathclyde, Glasgow, United Kingdom; New York University School of Medicine, United States of America

## Abstract

Human African trypanosomiasis (HAT), or sleeping sickness, results from infection with the protozoan parasites *Trypanosoma brucei (T.b.) gambiense* or *T.b.rhodesiense* and is invariably fatal if untreated. There are 60 million people at risk from the disease throughout sub-Saharan Africa. The infection progresses from the haemolymphatic stage where parasites invade the blood, lymphatics and peripheral organs, to the late encephalitic stage where they enter the central nervous system (CNS) to cause serious neurological disease. The trivalent arsenical drug melarsoprol (Arsobal) is the only currently available treatment for CNS-stage *T.b.rhodesiense* infection. However, it must be administered intravenously due to the presence of propylene glycol solvent and is associated with numerous adverse reactions. A severe post-treatment reactive encephalopathy occurs in about 10% of treated patients, half of whom die. Thus melarsoprol kills 5% of all patients receiving it. Cyclodextrins have been used to improve the solubility and reduce the toxicity of a wide variety of drugs. We therefore investigated two melarsoprol cyclodextrin inclusion complexes; melarsoprol hydroxypropyl-β-cyclodextrin and melarsoprol randomly-methylated-β-cyclodextrin. We found that these compounds retain trypanocidal properties *in vitro* and cure CNS-stage murine infections when delivered orally, once per day for 7-days, at a dosage of 0.05 mmol/kg. No overt signs of toxicity were detected. Parasite load within the brain was rapidly reduced following treatment onset and magnetic resonance imaging showed restoration of normal blood-brain barrier integrity on completion of chemotherapy. These findings strongly suggest that complexed melarsoprol could be employed as an oral treatment for CNS-stage HAT, delivering considerable improvements over current parenteral chemotherapy.

## Author Summary

Human African trypanosomiasis (HAT) is caused by infection with either *Trypanosoma brucei gambiense* or *T.b.rhodesiense* and is fatal if untreated. In the late stages of the disease the parasites enter the brain, producing severe neurological symptoms. The arsenical drug melarsoprol is the only treatment available for *rhodesiense* disease once it has reached the brain. Melarsoprol is very poorly soluble in water which severely limits its oral bioavailability. Currently melarsoprol is solubilised in propylene glycol which restricts its administration to the intravenous route and treatment schedules are protracted and extremely painful. Further, this formulation of melarsoprol is toxic and kills 5% of all treated patients through the induction of a severe post-treatment reactive encephalopathy. In this study we show that combining melarsoprol with cyclodextrin molecules increases the oral bioavailability of the drug. In a murine model of late stage HAT oral treatment regimens employing melarsoprol cyclodextrin inclusion complexes rapidly cleared parasites from the brain, restored blood-brain barrier function and reduced the severity of the neuropathological response induced by the infection. If complexed melarsoprol is equally efficacious in patients with HAT this would not only have significant positive socio-economic impact but also constitute a major therapeutic advance in the field.

## Introduction

Human African trypanosomiasis (HAT), also known as sleeping sickness, is endemic in 36 countries, in sub-Saharan Africa where 60 million people are at risk from infection [1], [2]. The disease is caused by the protozoan parasites *Trypanosoma brucei (T.b.) gambiense* in West Africa and *T.b.rhodesiense* in East Africa and is spread by the bite of the tsetse fly vector [1], [2]. Infection with *T.b.gambiense* usually results in a disease that follows a chronic course which can last for up to several years before death ensues while *T.b.rhodesiense* infection follows an acute pattern with death occurring in only weeks to months [3]. In both infections the disease progresses in two stages, the early or haemolymphatic stage and the late encephalitic or CNS-stage [3]. During the early-stage the parasites migrate from the site of the tsetse fly bite and spread throughout the body via the blood and lymph, invading the peripheral organs. The trypanosomes then cross the blood-brain barrier (BBB) and migrate into the CNS to cause the characteristic clinical manifestations of CNS-stage disease such as alteration of sleep patterns, neuropsychiatric symptoms and a variety of motor and sensory disturbances [4]. If the disease is diagnosed during the early stage it can be treated with pentamidine (for *T.b.gambiense*) or suramin (for *T.b.rhodesiense*) [5]. If the infection has reached the CNS, *T.b.gambiense* infections can be treated with either a concise 10-day regimen of melarsoprol [6], [7] or the recently developed nifurtimox-eflornithine combination therapy (NECT) [8]. In the case of CNS-stage *T.b.rhodesiense* infections the only treatment option currently available is a lengthy melarsoprol schedule comprising 3–4 cycles of a series of 3–4 injections, of increasing melarsoprol concentration, separated by a 7–10 day interval between each cycle [6], [9].

**Figure 1:**
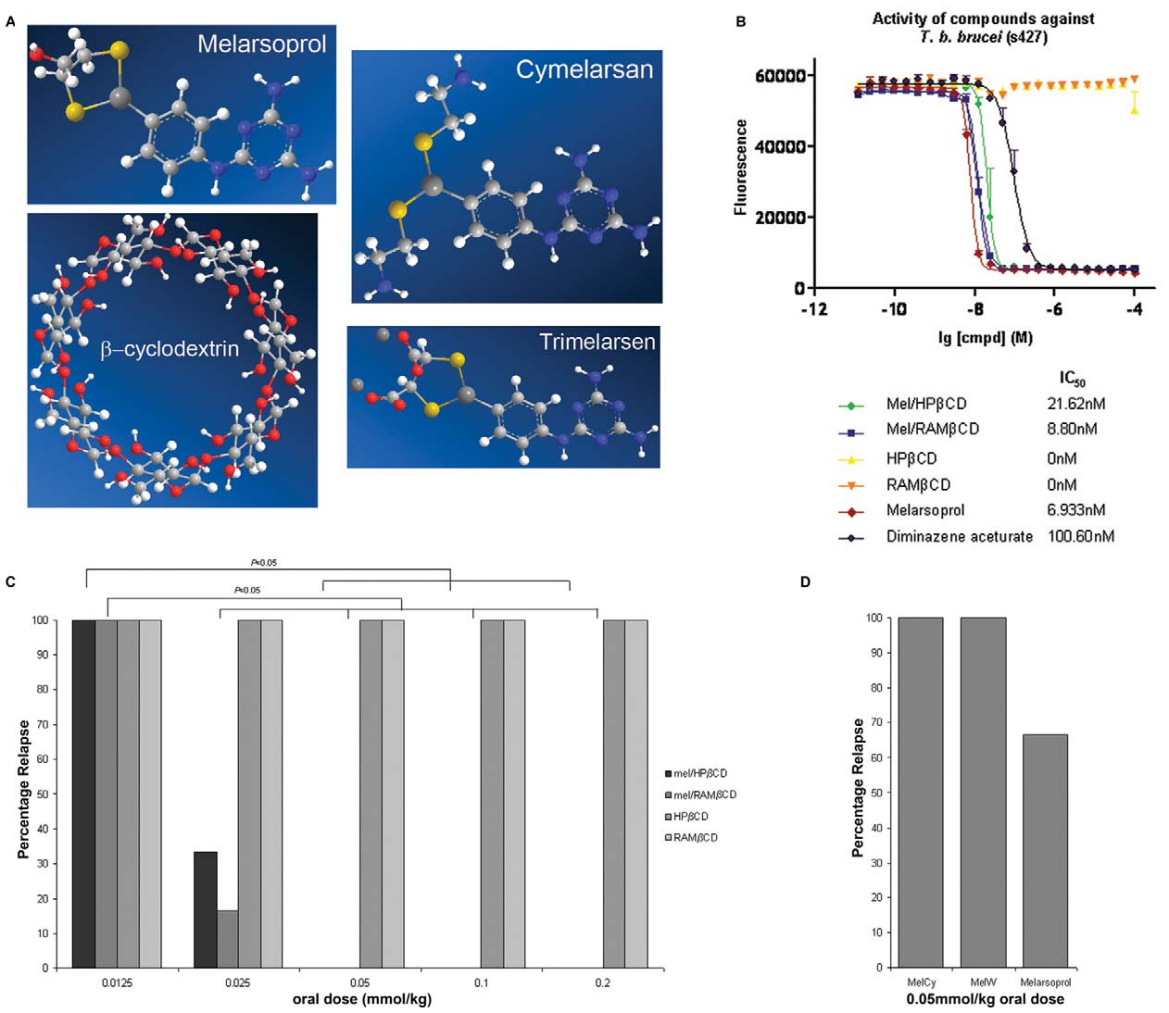
*In vitro and in vivo* efficacy of melarsoprol cyclodextrin inclusion complexes. The trypanocidal activity and the efficacy of oral administration of a variety of melarsoprol formulations was assessed. (A) Chemical structures of melarsoprol, MelCy (cymelarsan®) and MelW (trimelarsen®) are shown together with a β-cyclodextrin molecule used to form the melarsoprol inclusion complexes. (B) The IC_50_ values of mel/HPβCD and mel/RAMβCD were evaluated against *T.b.brucei* trypanosomes using an Alamar blue assay and compared with those of melarsoprol, diminazene aceturate and the empty cyclodextrin molecules. The assay was performed using duplicate samples on three independent occasions. The *in vitro* trypanocidal activity of the melarsoprol inclusion complex were no different to that of standard melarsoprol (*P* = 0.2002, *P* = 0.999; mel/HPβCD, mel/RAMβCD respectively). (C) The empty cyclodextrins and the melarsoprol cyclodextrin inclusion complexes were given to *T.b.brucei* infected mice daily for a period of 7-days by oral gavage beginning on day 21 post-infection during the CNS-stage of the disease (n = 6 per group). Relapse rates, drug dosages and significant differences between the efficacy of the dosages are shown. (D) Standard melarsoprol, MelCy and MelW, failed to produce satisfactory cure rates when administered following an identical dosing regimen to that of the inclusion complexes.

Melarsoprol (Figure 1A) is a highly lipophilic molecule that is poorly soluble in water with a log P_OW_ of 2.53 and a solubility of only 6 mg/L at 25°C [10]. Despite these properties the drug is a potent trypanocide and has been used for the treatment of HAT since its introduction in 1949 [11]. The limited solubility of melarsoprol precludes its oral delivery as only a small fraction of the drug is absorbed through the gastrointestinal tract. Currently melarsoprol is produced as a 3.6% solution in propylene glycol (Arsobal ®) which restricts its administration to the intravenous route. The treatment schedules employed are protracted, excruciatingly painful and require continuous hospitalization. In addition, treatment with Arsobal® is associated with numerous adverse effects including severe tissue necrosis at the injection site, neuropathy, and gastrointestinal upset [4]. However, the most serious adverse reaction is the development of a post-treatment reactive encephalopathy (PTRE) which occurs in 10% of all treated patients, 50% of whom die as a result. Arsobal® treatment is therefore responsible for the death of 5% of all patients given the drug [1], [12]. Although the pathogenesis of the PTRE remains unclear, several hypotheses have been postulated to explain its occurrence. These include direct arsenical toxicity [13], [14], autoimmune reactions [15] and pro-inflammatory immune response directed against trypanosomes remaining within the CNS following systemic clearance of the parasites [16], [17] or parasite antigen released as a consequence of chemotherapy [18]. The severity of the complications associated with Arsobal® chemotherapy make accurate staging of the disease via cerebrospinal fluid analysis absolutely essential both to manage proven CNS-stage infections appropriately and to prevent unnecessary administration of this highly toxic drug to early stage patients [19].

Cyclodextrins are naturally occurring cyclic oligosaccharide molecules composed of six or more glycopyranose units linked by α-1, 4 gycosidic bonds. They take the form of a truncated cone or torus with a hydrophilic exterior and a hydrophobic interior cavity which can be occupied by various guest molecules [20]. Cyclodextrins have been widely utilized by the pharmaceutical industry to alter the physiochemical properties of a variety of drugs through enhancing their solubility and oral bioavailability and decreasing their toxicity [10], [21]. Complexation of melarsoprol with either hydroxypropyl-β-cyclodextrin (mel/HPβCD) or randomly methylated-β-cyclodextrin (mel/RAMβCD) (Figure 1A) has been shown to increase the inherent solubility of the drug by a factor of 7.2×10^3^, which raises the possibility that the melarsoprol cyclodextrin complexes could be efficacious when delivered via the oral route for the treatment of trypanosomiasis [10].

In the current study the efficacy of the melarsoprol cyclodextrin inclusion complexes was investigated using both *in vitro* and *in vivo* methodologies and compared with that of contemporary melarsoprol formulations. The effect of oral drug treatment on the BBB was examined using MRI, and both the CNS parasite load and the CNS neuroinflammatory response monitored throughout the treatment regimen. We show here that melarsoprol cyclodextrin complexes are orally effective and non-toxic in curing CNS-stage trypanosome infections in mice.

## Methods

### Alamar blue assay

Trypanotoxicity was determined using an adapted version of the Alamar Blue assay [22]. Bloodstream form *T. brucei brucei* (strain 427) were cultivated in HMI-9 medium (BioSera Ltd., UK) [23] supplemented with 2 mM β-mercaptoethanol (Sigma-Aldrich, UK) and 10% fetal calf serum (BioSera Ltd., UK) at 37°C in a humidified 5% CO_2_ environment. Parasites (100 µl of 1×10^4^ trypanosomes/ml) were added to wells of 96-well plates containing doubling dilutions of the drugs (100 µl) ranging in final concentration from 100 µM to 24 pM and incubated for 48 hours. Alamar Blue reagent (20 µl, 0.49 mM in PBS, pH 7.4; Sigma-Aldrich, UK) was added to each well and, after 24 hours, the fluorescence was measured using a LS 55 luminescence spectrophotometer (PerkinElmer Life and Analytical Sciences, USA) set at excitation and emission wavelengths of 530 nm and 590 nm respectively. Data was analysed and inhibitory concentration (IC_50_) values determined with Prism 5.0 (GraphPad Software, USA) software. The experiment was performed in duplicate on three independent occasions.

### Animals and infections

A well established and characterised model of CNS-stage human African trypanosomiasis was employed throughout this investigation. Briefly, female CD-1 mice (Charles River Laboratories) (20–30 g body weight) were infected with 3×10^4^ *Trypanosoma brucei brucei* GVR35 parasites by intraperitoneal injection. The infection was allowed to progress until day 21 without drug intervention. At this point the parasites have established within the CNS and the infection has entered the encephalitic stage.

Infection was confirmed in all mice prior to drug treatment by examination of a wet blood film for the presence of parasites. To determine whether a treatment regimen was curative, blood smears were examined for the presence of parasites on a weekly basis for a period of 60 days. If the animals relapsed to parasitaemia the regimen was considered unsuccessful and the mice were killed. Mice that remained aparasitaemic for the duration of the monitoring period were killed, the brains excised and lightly homogenised in PBS supplemented with 1.5% glucose w/v and injected intraperitoneally into a clean recipient animal. This mouse was then monitored for the presence of parasites for a further 60 days. If the mouse remained aparasitaemic the treatment regimen was considered successful.

All animal procedures were authorised under the Animals (Scientific Procedures) Act 1986 and approved by the University of Glasgow Ethical review Committee.

### Quantitative PCR

Trypanosome load within the brain was determined by real-time quantitative PCR. Briefly, whole brains were homogenised and digested with proteinase K (AppliChem GmbH) and DNA extracted from a 25 mg sample of the homogenate (Qiagen, DNeasy Tissue kit). The concentration of the extracted DNA was assessed by measuring the absorbance and the sample diluted to 20 ng/ml. The reaction mix was comprised of; Taqman Brilliant II master mix (Agilent), 0.05 pmol/µL of each primer, 0.1 pmol/µL probe (Eurofins MWG Operon) and 100 ng DNA template. The amplification was performed on a MxPro 3005 thermocycler (Agilent).

### Drugs and treatment regimens

The mel/HPβCD and mel/RAMβCD inclusion complexes were prepared as previously described [10]. Each complex was dissolved in sterile water and administered at dose rates of 0.0125, 0.025, 0.05, 0.1 and 0.2 mmol/kg (equivalent to 4.975, 9.95, 19.9, 39.8, and 79.6 mg/kg) of the active ingredient, melarsoprol. Non-complexed HPβCD and RAMβCD (Sigma) were used as control treatments and administered at dose rates equivalent to 0.1 mmol/kg of the complexed agent.

Contemporary melarsoprol and the melaminophenyl arsine derivatives [24], melarsamine hydrochloride (MelCy) (Cymelarsan®) and melarsonyl potassium (MelW) (Trimelarsen®) were prepared as solutions or fine suspensions in sterile water and administered at a dose of 0.05 mmol/kg.

All drug treatments were delivered orally by gavage, once per day for a period of 7 days beginning on day 21 post-infection.

Body weights were measured in groups of uninfected mice before and after completion of treatment and clinical appearance was monitored using an established visual assessment scale [25] throughout the drug regimens to assess overt signs of drug toxicity.

### Magnetic Resonance Imaging

MRI was performed on two mice at day 21 post-infection prior to drug treatment. The mice were re-scanned at 24 hours, 8 and 15 days after completion of chemotherapy. Uninfected mice (n = 3) were also examined. All scans were performed as described previously [26]. Briefly, mice were anaesthetised and the tail vein was cannulated with a 26 gauge×19 mm cannula to facilitate contrast agent administration during MRI scanning. The animal was placed into a mouse cradle and restrained using ear and tooth bars to minimise head movement. Anaesthesia was maintained throughout the procedures and respiration, heart rate and body temperature were observed. The animal was maintained normothermic by an enclosed warm water circuit.

MRI was performed on a Bruker Biospec 7 T/30 cm system equipped with an inserted gradient coil (121 mm ID, 400 mT/m) and a 72 mm birdcage resonator. A surface coil was used for brain imaging. The scanning protocol consisted of a RARE T_2_ weighted scan [effective TE (echo time) 76 ms, TR (repetition time) 5362 ms, 25 averages, matrix 176×176, FOV (field of view) 17.6×17.6 mm, 20 contiguous coronal slices of 0.4 mm thickness] followed by a RARE T_1_ weighted scan (effective TE 9 ms, TR 8000 ms, 20 averages, matrix 176×176, FOV 17.6×17.6 mm, 20 contiguous coronal slices of 0.4 mm thickness). Following the RARE T_1_ weighted scan 0.1 ml of a solution containing 50 µL gadolinium-diethylenetriamine penta-acetic acid (Gd-DPTA Magnevist®; Bayer) and 50 µL of sterile water was injected via the tail vein cannula. Five minutes later the T_1_ weighted scan was repeated. Gd-DTPA cannot readily cross the intact blood brain barrier due to its charge and high molecular weight [27]. Extravasation of Gd-DTPA observed within the parenchyma demonstrates an impairment of the BBB integrity.

Images were analysed using Image J software (http://rsbweb.nih.gov/ij/). Contrast enhancement maps were generated from the the per and post-contrast T_1_ weighted scans according to the equation: *Enh = (S_post_−S_pre_)÷S_pre_* where *S_post_* = post contrast agent signal and *Spre* = pre-contrast agent signal. Regions of interest (ROIs) were manually defined to include the entire brain and meninges. The mean percentage signal change for each brain slice was then calculated and signal enhancement maps generated.

### Histopathological analysis

Following sacrifice the brains were excised and fixed in 4% neutral buffered formalin, paraffin wax blocks prepared and sections of 3 µm thickness cut and stained with haematoxylin and eosin. These sections were examined by two independent assessors and the severity of the neuropathological reaction graded on a scale of 0–4 where 0 represented normal pathology with no indications of inflammation and a grade of 4 was characterised by the presence of a severe meningoencephalitis with the presence of inflammatory cells in the brain parenchyma [26], [28] (Table S5).

Immunocytochemistry was performed to detected T-cells (rabbit anti-CD3), B-cells (rat anti-B220) and macrophages (rat anti-F4/80) following a standard peroxidise anti-peroxidase protocol using the Dako® EnVision system and DAB visualisation.

### Statistical analyses

Data were analyzed using analysis of variance methods and the General Linear Model (GLM) procedure in Minitab Version 16 followed by multiple pair wise comparison tests. This identified significant main effect differences between groups of uninfected animals, infected animals and treated animals. In studies with measurements over time the GLM procedure provided tests for treatment and time effects and their interaction. Proportions of mice relapsing in different treatment groups were compared using a Tukey-type multiple comparison test for proportions [29]. Changes in body weight were investigated using a paired t-test. P values of less than 5% were considered to be statistically significant. Where appropriate data were log transformed prior to analysis. Group means were plotted showing means and their standard errors, and the size of treatment effects were estimated using differences between group means and their 95% confidence intervals. Log dose response curves provided estimates of IC_50_ concentrations.

## Results

### Assessing the trypanocidal activity of melarsoprol cyclodextrin inclusion complexes

To determine whether the complexed melarsoprol retains its trypanocidal properties a modification of the Alamar blue assay [22] was used to investigate the inhibitory concentration (IC_50_) of the complexed melarsoprol molecules in comparison to standard melarsoprol and an unrelated trypanocidal drug, diminazene aceturate, in an *in vitro* trypanosome culture system. The IC_50_ values determined for mel/HPβCD and mel/RAMβCD were 21.6 nM and 8.8 nM respectively (Figure 1B). Standard melarsoprol returned an IC_50_ value of 6.9 nM. Statistical analyses of the Alamar blue data revealed no significant changes in the trypanocidal activity of melarsoprol following complexation when compared to the standard form of the drug (*P* = 0.2002, *P* = 0.9999; mel/HPβCD and mel/RAMβCD respectively). The HPβCD and RAMβCD molecules alone did not display any trypanocidal activity (Table S1, Figure 1B).

### Determining the efficacy of melarsoprol cyclodextrin inclusion complexes *in vivo*

The ability of the complexed melarsoprol compounds to cure CNS-stage trypanosome infections was investigated in a well established and characterized murine model of the disease. The drugs were administered by oral gavage each day at doses ranging from 0.0125 mmol/kg to 0.2 mmol/kg for a seven day period. All animals became aparasitaemic following the melarsoprol regimens; however, all mice treated at the 0.0125 mmol/kg level relapsed to parasitaemia. A relapse to parasitaemia was also detected in one third of the mice treated with mel/HPβCD and one sixth of the mice given mel/RAMβCD at the 0.025 mmol/kg level. Successful cures were obtained in all mice treated with the 0.05 mmol/kg, 0.1 mmol/kg or 0.2 mmol/kg dosage of either complex, indicating that 0.05 mmol/kg was the minimum dosage necessary to achieve successful cures. Animals given HPβCD or RAMβCD alone remained parasitaemic throughout the procedure (Figure 1C).

Paired t-test analysis detected no evidence of decreased body weight in uninfected mice following 7-days of oral drug treatment. A significant (*P* = 0.019, 95% confidence interval 0.213 g, 1.954 g) increase was detected between the mean body weight of the pre- and post treatment groups (25.83±0.696 g; 26.92±0.890 g respectively). No adverse clinical signs were detected at any point during the chemotherapy regimen with the mice remaining alert and showing good coat condition.

### Determining the efficacy of contemporary melarsoprol formulations *in vivo* following oral administration

The efficacy of melarsoprol (MelB) and the water soluble melaminophenyl arsine derivatives [24], melarsamine hydrochloride (MelCy) and melarsonyl potassium (MelW) (Figure 1A) when administered *per os* at a dose of 0.05 mmol/kg for seven consecutive days, during CNS-stage infections was investigated. No cures were obtained in the mice treated with MelCy or MelW and only 33% of the mice given MelB were successfully cured (Figure 1D).

### Assessing parasite load within the brain following oral complexed melarsoprol treatment

**Figure 2:**
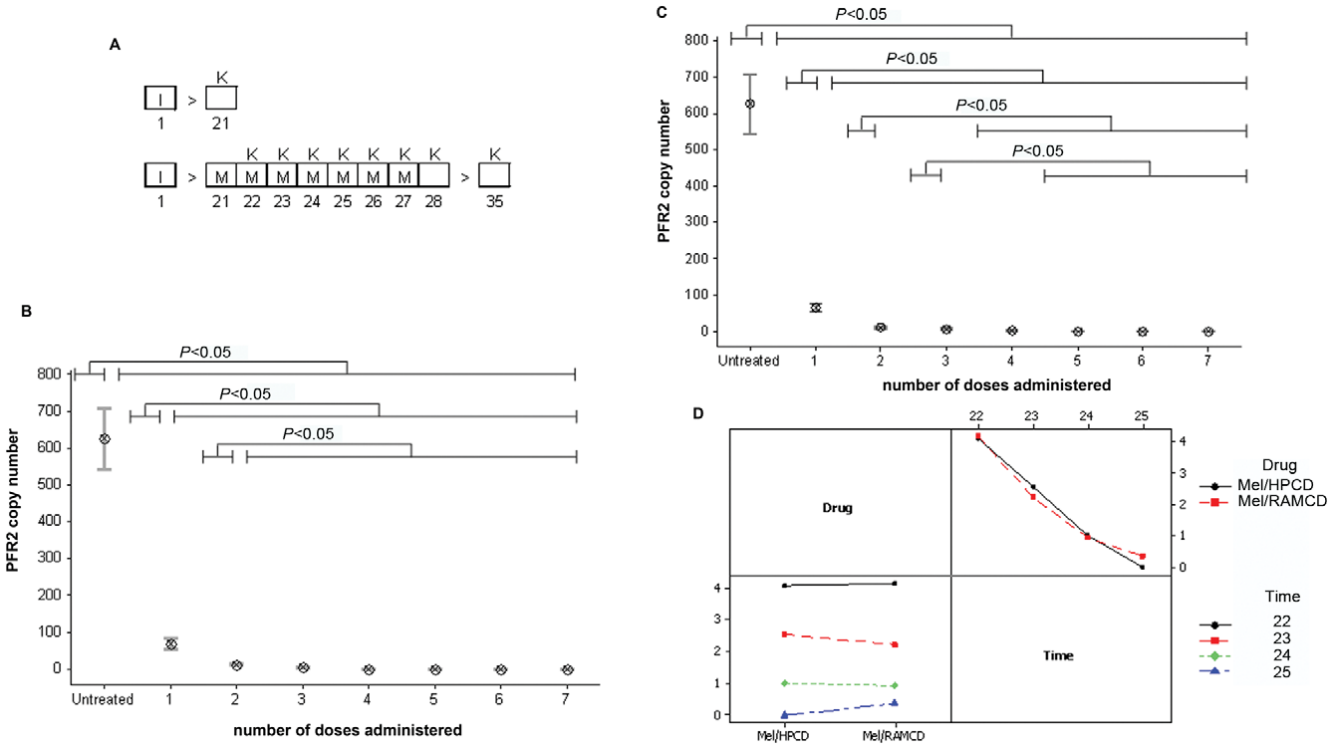
Clearance of parasites from the brain following mel/HPβCD or mel/RAMβCD chemotherapy. The ability of the melarsoprol cyclodextrin inclusion complexes to clear the trypanosomes form the brain was studied. (A) Animals were infected (I) with *T.b.brucei*. On day 21 post-infection one group (n = 6) of mice were sacrificed (K) while the remainder were treated with mel/HPβCD or mel/RAMβCD (M) at 0.05 mmol/kg. Further groups (n = 6) were sacrificed 24 hours following each drug administration. Days post-infection are indicated below the treatment schedule. Trypanosome load within the brain was assessed using Taqman PCR (Figure S1); (B,C) Interval plots of trypanosome load following each drug administration showing the mean and standard error of the mean are presented for mel/HPβCD and mel/RAMβCD respectively. Analyses of the data using the GLM procedure identified significant differences between the means (Table S2 & S3). (D) Interaction plot demonstrates no significant interaction between mel/HPβCD and mel/RAMβCD treatment and the trypanosome load after each administration (*P* = 0.813).

Taqman real-time PCR was performed (Figure S 1) to determine the parasite numbers present within the brain tissue prior to chemotherapy and at 24 hours after each oral dose of mel/HPβCD or mel/RAMβCD (Figure 2A). Animals killed on day 21 post-infection, prior to receiving any drug treatment showed a mean parasite load of 626±82.8 (mean ± SE). Following a single dose of mel/HPβCD or mel/RAMβCD the parasite numbers detected within the brain were significantly (*P*<0.001) reduced (68.1±14.7; 66.2±10.8 respectively). The decrease in parasite numbers continued in a stepwise manner with successive treatments until the trypanosomes were completely cleared from the brain (Figure 2B & C, Table S2 & S3). Interaction plots comparing the mean copy numbers detected after each dose of mel/HPβCD and mel/RAMβCD show that there are no significant differences between the clearance rates achieved by either of the drugs (Figure 2D). From the Taqman results it is apparent that both forms of complexed melarsoprol clear the trypanosomes from the brain in a rapid and efficient manner with a reduction of greater than 80% of the parasite load 24 hours after the initial drug treatment.

### MRI of BBB intergrity

**Figure 3:**
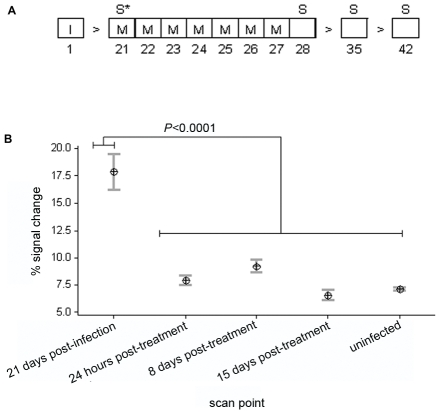
MRI scanning regimen and evaluation of blood-brain barrier integrity. The effect of mel/HPβCD treatment on the integrity of the blood-brain barrier was assessed using MRI. (A) Treatment and scanning scheduled employed. Infected (I) mice (n = 2) were treated with mel/HPβCD at 0.05 mmol/kg (M) and scanned (S) at the times indicated and prior to the initial drug treatment (S*). (B) Interval plot, showing the mean and standard error of the mean, of MRI post-contrast percentage signal change data at each scan point. GLM analyses identified significant differences between the group means (Table S4).

**Figure 4:**
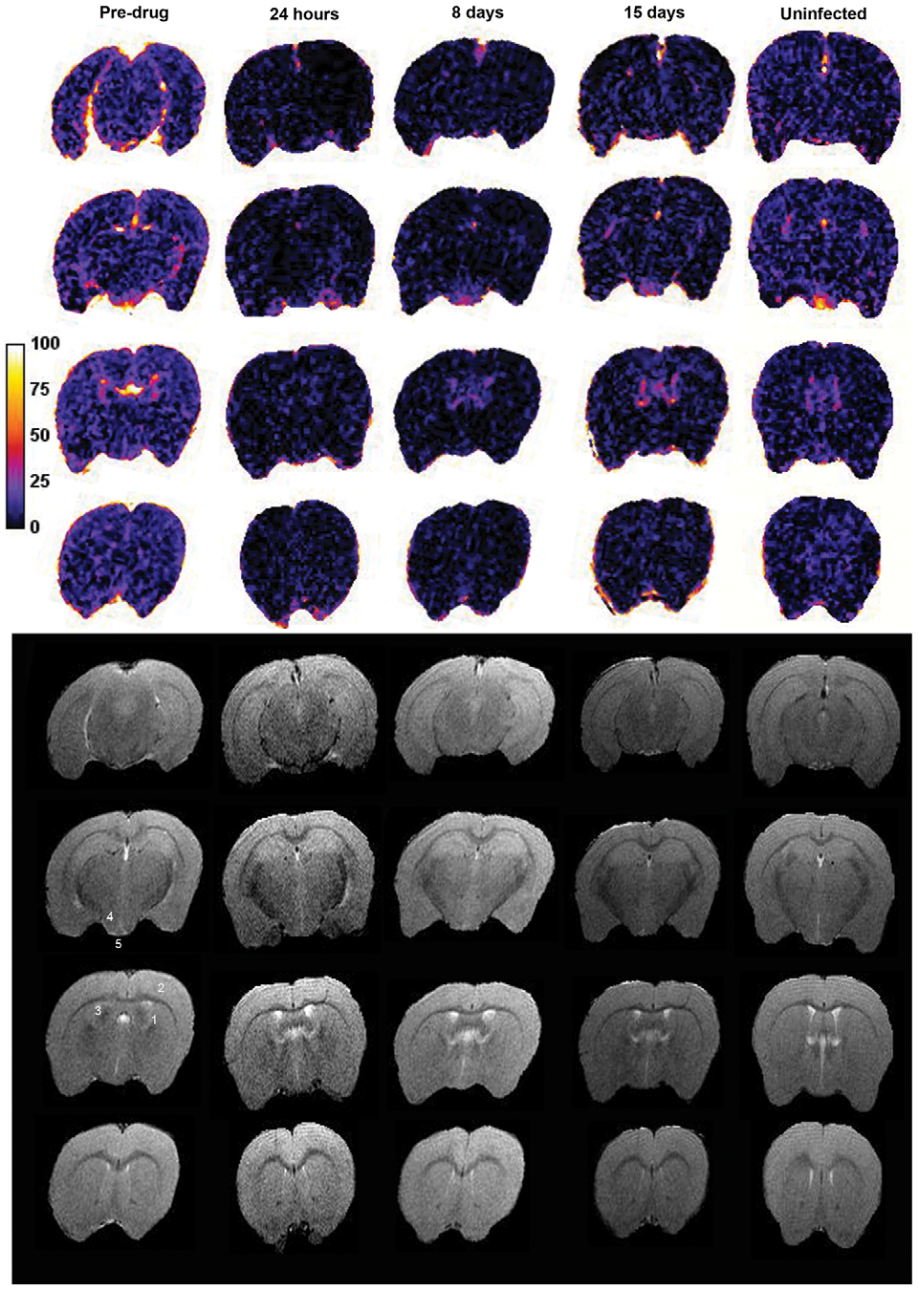
Serial MRI scans prepared from each time point investigated. The signal enhancement maps (top) and T2-weighted images (bottom) of 4 representative slices (hind brain to fore brain) from an animal serially scanned prior to drug treatment, 24 hours, 8 days, and 15 days after completion of the treatment course as well as an uninfected control mouse are shown. Brian areas including the ventricular region (1), cerebral cortex (2), hippocampus (3), hypothalamus (4) and median eminence (5) are representatively labeled on pre-drug treated T-2 images.

We determined the effect of oral treatment with mel/HPβCD on BBB function using MRI. Mice were examined prior to treatment, and 24 hours, 8 and 15 days following the chemotherapy regimen (Figure 3A). MRI scans were performed before and after the injection of Magnevist® contrast agent (Gd-DPTA) [27] and signal enhancement maps generated as previously described [26]. Changes in BBB integrity were investigated in two infected mice scanned at day 21 post-infection and compared with scans prepared in the same animals 24 hours, 8 days and 15 days after completing a 7 day oral course of mel/HPβCD as well as those from uninfected mice (n = 3). At day 21 post-infection the BBB was significantly compromised (17.87±1.62) (Figure 3B, Figure 4, Table S4). Signal enhancement was present throughout the brain with highest signal change found in the ventricular region. Changes in signal intensity were also apparent in the cerebral cortex, hypothalamus, hippocampus and median eminence (Figure 4). However, by 24 hours after completion of the chemotherapy (Figure 4) the percentage signal change (7.93±0.455) had dropped significantly (*P*<0.0001) and was comparable (*P* = 0.9296) to that seen in uninfected mice (7.11±0.162) (Figure 3B, Figure 4) indicating that by this point the integrity of the BBB had become re-established. The integrity of the barrier was maintained in all subsequent scans performed at 8 days (9.25±0.596) (Figure 3B, Figure 4) and 15 days (6.55±0.463) (Figure 3B, Figure 4) after completion of the treatment schedule (Table S4).

### Assessing the severity of the neuropathological response

**Figure 5:**
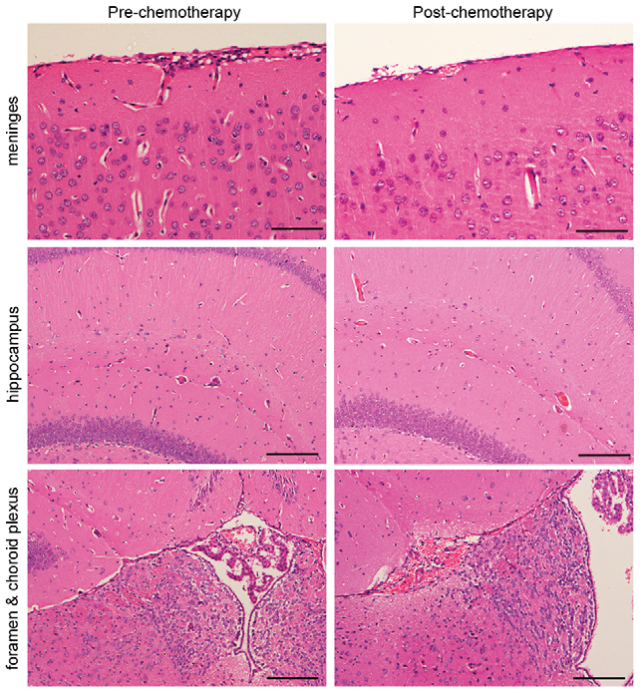
Histopathology demonstrating the neuropathological response in mice. Sections are prepared from mice killed at day 21 post-infection, prior to drug treatment (left column) and animals killed 15 days after completion of the treatment with mel/HPβCD (right column). H&E stained coronal sections showing the neuropathological response in the meninges, hippocampus and choroid plexus/interventricular foramen (scale bars from top 100 µm, 200 µm and 200 µm).

**Figure 6:**
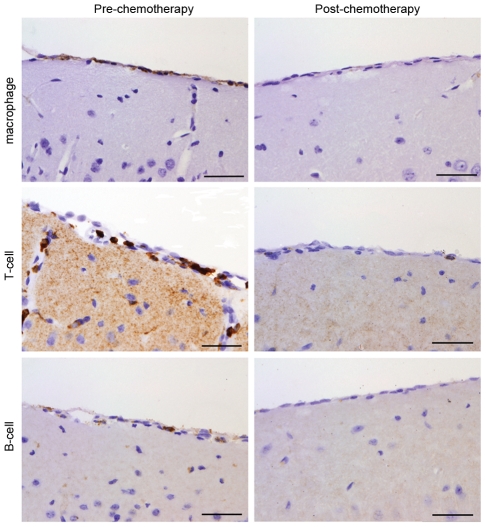
Immunocytochemistry demonstrating macrophages, T-cells and B-cells. Macrophages, T-cells and a few B-cells were present within the inflammatory cell infiltrate in mice prior to drug treatment (left column). Following drug treatment these cell types were rarely seen (right column) (scale bars 50 µm).

The severity of the neuropathological response to the trypanosome infection and drug treatment was determined in mice killed 15 days after completing the treatment schedule and compared to animals killed at day 21 post-infection prior to receiving chemotherapy using a well established grading scale [28] (Table S5). Pathological examination of the brains prepared from animals prior to drug treatment showed mild neuroinflammatory changes (1.5±0.158) with the presence of an inflammatory cell infiltrate in the meninges and Virchow–Robin spaces (Figure 5). Some perivascular cuffs were also apparent surrounding the blood vessels in the hippocampus (Figure 5). The cellular infiltrate was composed mainly of lymphocytes, and macrophages (Figure 6). A significant (*P* = 0.0366) resolution of this neuroinflammation (1.083±0.083) was apparent in mice killed 15 days after completion of the oral mel/HPβCD regimen. This represents a mean decrease of 27.8% with a 95% confidence interval (0.032, 0.801). Only a few inflammatory cells could be detected in the meninges of these animals accompanied by very mild perivascular infiltration of the occasional blood vessel in the hippocampus (Figure 5 & 6).

## Discussion

New drugs to treat HAT remain an urgent priority. In spite of some recent positive developments [30] the situation remains precarious as evidenced by the failure, late in clinical trials, of pafuramidine (DB289). Ideally new drugs should be orally available and of equal or better efficacy than current drugs with improved safety. Melarsoprol is the only drug suitable to treat CNS-stage *rhodesiense* disease and remains in use in some areas for *gambiense*. Its use, however, is tainted by its being administered parenterally and through its well documented adverse events. The study reported here shows that complexation of melarsoprol with the cyclodextrin molecules does not affect the trypanocidal properties of the compound and appreciably enhances the ability of the drug to cure CNS-stage trypanosome infections when given as an oral chemotherapy regimen. The improved oral bioavailability seen in these investigations is most likely due to the increased solubility and reduced degradation kinetics of the drug following complexation with the cyclodextrin molecules [10], [31]. Further, cyclodextrins can also act as carriers, delivering the drug directly to the intestinal membrane while protected within the cavity of the molecule [32], [33]. Consistent with our findings is the improved oral bioavailability reported with a variety of cyclodextrin drug inclusion complexes including anti-parasitic agents. The potent anti-malarial drug artemisinin has low aqueous solubility that severely limits its absorption following oral administration. Complexation of artemisinin with cyclodextrin molecules has been shown to improve the pharmacokinetic profile of the drug compared with Artemisinin 250® when given *per os* [34]. This has also been demonstrated for the anti-helminthic drug albendazole [35].

The pathogenesis of the PTRE associated with standard melarsoprol treatment is currently unknown although several hypotheses have been suggested [13]–[18]. However, it is probably caused, at least in part, by an acute toxic reaction to low levels of arsenic within the CNS following delivery of an intravenous bolus of the arsenical drug [13], [14]. On the basis of our combined data the lack of toxicity and the resolution of the CNS inflammatory reaction shown following oral treatment with melarsoprol cyclodextrin inclusion complexes can most easily be explained by the more controlled delivery of the trypanocidal drug to the brain following a sustained absorption from the gut compared with that of an intravenous bolus. Consistent with this explanation are the extremely low levels of arsenic in the brain tissue following chemotherapy which were below the level of detection (<5 ng/mL) of the GC-MS assay employed (unpublished data) and the extremely rapid clearance of the parasites from the CNS following drug administration. This is also reflected by the restoration of BBB integrity detected in the mice 24 hours after completion of the chemotherapy regimen. However, a direct comparison of these criteria following a curative IV regimen of Arsobal® would be required to corroborate this hypothesis. Multiple IV doses of Arsobal® cannot be administered in the murine model due to the severe venous damage caused by the propylene glycol solvent present in the drug preparation. Therefore, data regarding drug levels, parasite clearance and BBB function following IV Arsobal® remain unavailable.

Taken together these findings strongly suggest that mel/HPβCD and mel/RAMβCD could be used to treat patients with CNS-stage HAT. Since these experiments were performed using a *T.b.brucei* model of infection it is possible that these drug complexes will not show the same activity profile when transferred to human disease. However, since the active trypanocidal component of the complex is melarsoprol, with proven effectiveness against both *T.b.rhodesiense* and *T.b.gambiense* infections, this scenario seems highly unlikely. Consequently, in the first instance, these complexes should be tested in subjects with *T.b.rhodesiense*, even though these comprise the minority of cases of HAT compared with *T.b.gambiense*, since melarsoprol is currently the *only* drug that can be effective in *rhodesiense* disease. The drugs are effective orally at dosages that could be delivered in humans. During the concise 10-day schedule for Arsobal® treatment a 60 kg patient would be given a total dose of 1320 mg of melarsoprol. In the current study, melarsoprol cyclodextrin complexes were curative when administered at 0.05 mmol/kg or 19.9 mg/kg melarsoprol daily for a seven day period. To obtain an approximate human equivalent dose (HED) from this data the dosage must be normalized according to body surface area which can be achieved by dividing the murine dose by a factor of 12 [36]. The HED for the complexed drugs would therefore be approximately 1.6 mg/kg, with a total dosage of 672 mg assuming a seven day course and a 60 kg body weight. This is a considerable reduction in the total amount of arsenical required for each drug course, even when compared with the concise schedule. This decreased arsenical dosage could also be a major factor in the apparent lack of toxicity associated with the oral regimen.

These complexes rapidly clear the trypanosomes from the brain following administration, reduce the severity of the neuropathological response induced by the infection, and also restore BBB integrity following treatment. The availability of an orally administrable drug would preclude both the need for hospitalization of the patient throughout the period of treatment and the provision of highly skilled clinicians to administer the drug by slow intravenous infusion. Further, the pain and fear associated with current melarsoprol therapy would be circumvented and patients would be far more likely to be compliant in finishing the treatment course. This would have a significant positive socio-economic impact in local communities and on the already burdened health care budgets of these regions.

One of the major problems in the management of HAT is that there is no clear consensus on the criteria used to classify an infection as having progressed to the CNS-stage [19], [37]. The current WHO criteria suggest that CSF containing >5 white blood cells (WBC)/µL with or without the presence of trypanosomes indicates CNS-stage infection [9]. However in some *T.b.gambiense* infections the higher value of >20 WBC/µL has been used before commencing melarsoprol treatment [38], [39]. This has significant implications for choosing the correct chemotherapeutic approach to best manage the infection. Inappropriate administration of melarsoprol to patients with early-stage disease exposes them to unnecessary risks form drug toxicity while failure to use melarsoprol in CNS-stage disease will have inevitably fatal consequences [19]. The use of an alternative treatment strategy without the associated adverse safety profile of the intravenous melarsoprol formulation would also obviate significantly the difficulties associated with the current methods of disease staging [19].

In conclusion, the current chemotherapy options for treatment of CNS-stage HAT are extremely limited and all involve parenteral administration of highly toxic and sometimes ineffective drugs. Moreover there are no new alternative drugs for CNS HAT likely to be used in clinical practice for at least 5–10 years [30]. Only one compound, fexinidazole, is currently in Phase I clinical trials [40]. Due to the high failure rate of novel compounds it is critical to maintain drug development in this area to ensure that effective treatments for both forms of this disease are available in the future. Sir James Black, the Nobel Laureate said ‘the most fruitful basis for the discovery of a new drug is to start with an old drug’ [30]. If melarsoprol cyclodextrin inclusion complexes, given via the oral route, prove equally efficacious in patients with HAT this would constitute one of the most significant therapeutic advances in the long history of the disease. Plans to test these drug complexes in a phase II trial in East African patients with *T.b.rhodesiense* are currently being formulated.

## Supporting Information

Figure S1 **Taq man analyses to determine parasite load.** Examples of the amplification plot and standard curves obtained using the primer and probe sets detailed for detection of the PFR2 gene to determine parasite load. Amplification was performed on an Agilent MxPro3005 thermocycler using Brilliant II mastermix (Agilent), 0.05 pmol/µL primer, 0.1 pmol/µL probe (labelled with FAM and TAMRA) and 100 ng template DNA.
(DOC)

Table S1 **Inhibitory concentration (IC_50_) of mel/HPβCD, mel/RAMβCD, melarsoprol, diminazene aceturate, HPβCD and RAMβCD.** The IC_50_ of each compound was determined against wild type S427 *T. b. brucei* trypanosomes by Alamar blue assay. The figures in the body of the table demonstrate the comparisons, in terms of statistical significance, between the IC_50_ (nM) of each compound, shown in the row and column headings. The p-values and 95% confidence intervals for differences are based on analysis using the logarithmic transformation [log(x+1)] of the IC_50_. The mean IC_50_ value ± the standard error and the number of repeats are also shown.
(DOC)

Table S2 **Copy number of the PFR2 gene detected within the brain following mel/HPβCD chemotherapy.** Mice were infected with *T. b. brucei* GVR35/C1.9. Mel/HPβCD chemotherapy commenced on day 21 post-infection. The compound was administered by orally gavage, daily for 7 consecutive days at dose of 0.05 mmol/kg. The number of copies of the PFR2 gene present within 100 ng of DNA prepared from approximately 25 mg of whole brain homogenate, 24 hours after administration of each dose was determined by Taqman PCR. The figures in the body of the table demonstrate the comparisons, in terms of statistical significance, between the number of copies of the PFR2 gene detected after administration of each dose, shown in the row and column headings. The p-values and 95% confidence intervals for differences are based on analysis using the logarithmic transformation [log(x+1)] of the copy number. The mean copy number ± the standard error and the number of animals per group are also shown. No copies of the PFR2 gene were detected following the 4^th^ treatment or subsequent drug doses these groups have been removed from the analysis.
(DOC)

Table S3 **Copy number of the PFR2 gene detected within the brain following mel/RAMβCD chemotherapy.** Mice were infected with *T. b. brucei* GVR35/C1.9. Mel/RAMβCD chemotherapy commenced on day 21 post-infection. The compound was administered by orally gavage, daily for 7 consecutive days at dose of 0.05 mmol/kg. The number of copies of the PFR2 gene present within 100 ng of DNA prepared from approximately 25 mg of whole brain homogenate, 24 hours after administration of each dose was determined by Taqman PCR. The figures in the body of the table demonstrate the comparisons, in terms of statistical significance, between the number of copies of the PFR2 gene detected after administration of each dose, shown in the row and column headings. The p-values and 95% confidence intervals for differences are based on analysis using the logarithmic transformation [log(x+1)] of the copy number. The mean copy number ± the standard error and the number of animals per group are also shown. No copies of the PFR2 gene were detected following the 5^th^ treatment or subsequent drug administration these groups have been removed from the analysis.
(DOC)

Table S4 **Comparison of the percentage signal change data generated from MRI scans.** Mice were infected with *T. b. brucei* GVR35/C1.9. Immediately prior to treatment commencing on day 21 post-infection, animals were MRI scanned. Following recovery from the MRI procedure animals were administered mel/HPβCD orally at a dose of 0.05 mmol/kg. Mel/HPβCD treatment continued for the next 6 days. Twenty-four hours, 8 and 15 days after administration of the last dose, corresponding to days 28, 35 and 42 post-infection respectively, the MRI scans were repeated. Each MRI scan consisted of 20 continuous coronal slices. The brain was manually selected in each slice and the percentage signal change calculated. The figures in the body of the table demonstrate the comparisons, in terms of statistical significance, between the times post-treatment shown in the row and column headings. The *P*-values and 95% confidence intervals are based on analysis using the percentage signal change for each slice. The mean signal change ± the standard error and the number of animals per group are also shown.
(DOC)

Table S5 **Parameters defining the injury score allocated to the severity of the neuropathology.** Injury sores are given horizontally while the criteria used to define the scores are detailed vertically.
(DOC)
