# Supplementary material for: Melarsoprol Cyclodextrin Inclusion Complexes as Promising Oral Candidates for the Treatment of Human African Trypanosomiasis
Source: PLoS Negl Trop Dis. 2011 Sep 6;5(9):e1308. doi: 10.1371/journal.pntd.0001308 (PMC3167784; doi:10.1371/journal.pntd.0001308)
Supplement: Figure S1 — Taq man analyses to determine parasite load. Examples of the amplification plot and standard curves obtained using the primer and probe sets detailed for detection of the PFR2 gene to determine parasite load. Amplification was performed on an Agilent MxPro3005 thermocycler using Brilliant II mastermix (Agilent), 0.05 pmol/µL primer, 0.1 pmol/µL probe (labelled with FAM and TAMRA) and 100 ng template DNA. (DOC) [file pntd.0001308.s001.doc]

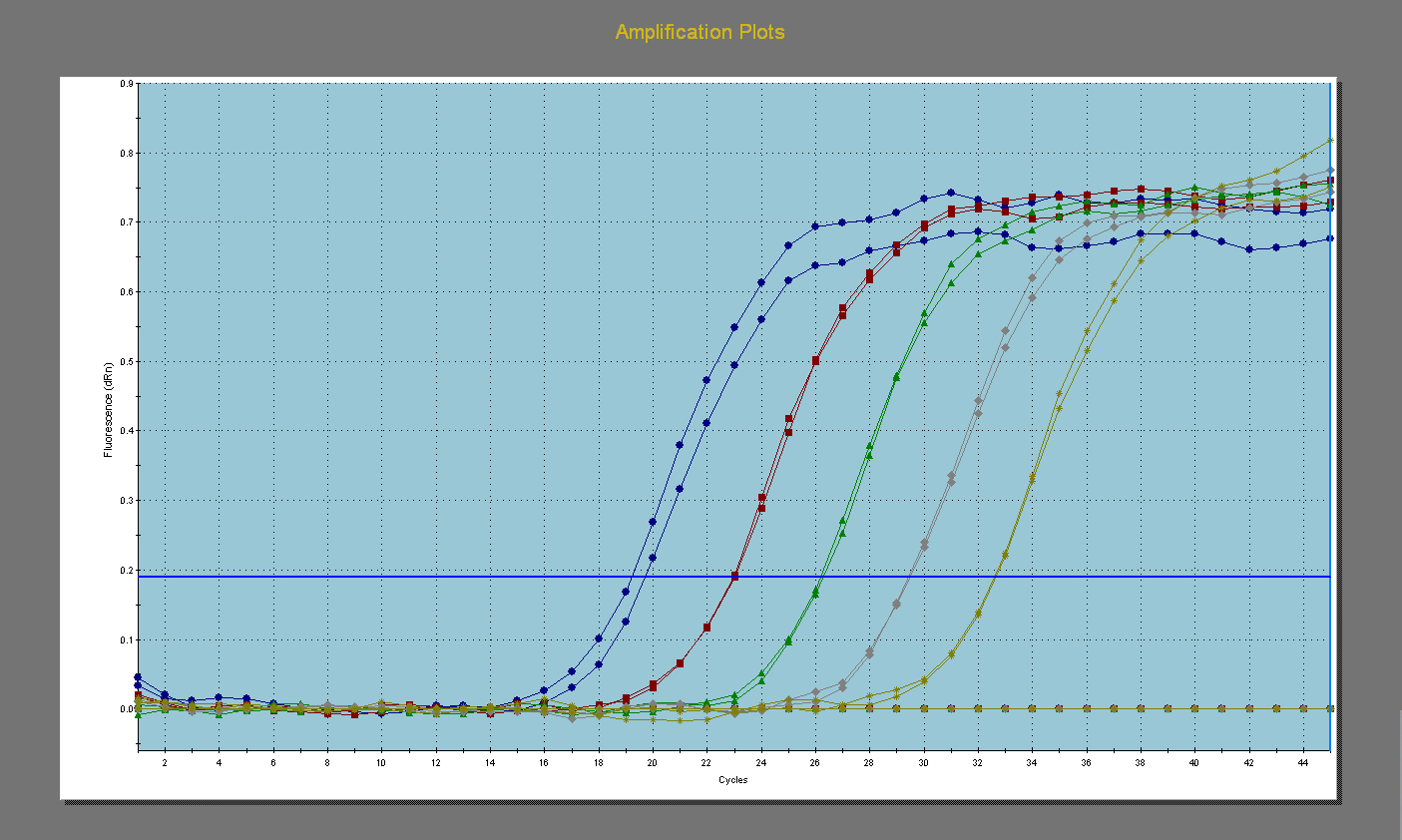

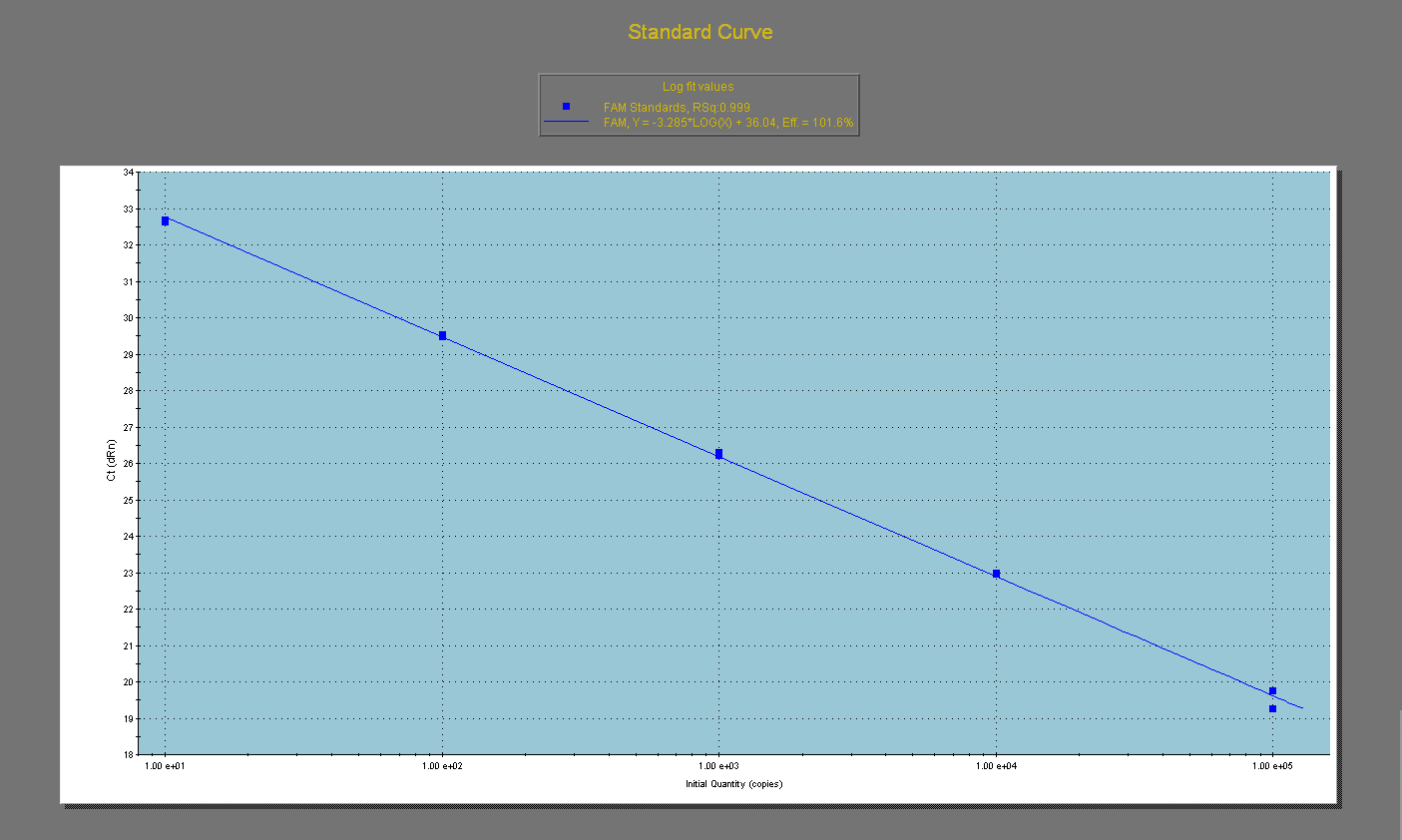


Log fit values

FAM standards, RSq:0.999

FAM, Y=-3.285*LOG(X)+36.04

Eff. =101.6%

**Amplification Plot**

**Standard Curve**

| Gene | Primers | Probe |
| --- | --- | --- |
|  | FW - _2563_ccaaccgtgtgtttcctcct_2583_  RV - _2636_gaaaaggtgtcaaactactgccg_2656_ | _2603_cttgtcttctccttttttgtctctttccccct_2634_ |

**Figure S1. Taq man analyses to determine parasite load.**

Examples of the amplification plot and standard curves obtained using the primer and probe sets detailed for detection of the PFR2 gene to determine parasite load. Amplification was performed on an Agilent MxPro3005 thermocycler using Brilliant II mastermix (Agilent), 0.05pmol/μL primer, 0.1pmol/μL probe (labelled with FAM and TAMRA) and 100ng template DNA.
