# Supplementary material for: Melarsoprol Cyclodextrin Inclusion Complexes as Promising Oral Candidates for the Treatment of Human African Trypanosomiasis
Source: PLoS Negl Trop Dis. 2011 Sep 6;5(9):e1308. doi: 10.1371/journal.pntd.0001308 (PMC3167784; doi:10.1371/journal.pntd.0001308)
Supplement: Table S1 — Inhibitory concentration (IC50) of mel/HPβCD, mel/RAMβCD, melarsoprol, diminazene aceturate, HPβCD and RAMβCD. The IC50 of each compound was determined against wild type S427 T. b. brucei trypanosomes by Alamar blue assay. The figures in the body of the table demonstrate the comparisons, in terms of statistical significance, between the IC50 (nM) of each compound, shown in the row and column headings. The p-values and 95% confidence intervals for differences are based on analysis using the logarithmic transformation [log(x+1)] of the IC50. The mean IC50 value ± the standard error and the number of repeats are also shown. (DOC) [file pntd.0001308.s002.doc]

Table S1: Comparison of the inhibitory concentration (IC_50_) of the mel/HPβCD, mel/RAMβCD, melarsoprol, diminazene aceturate, HPβCD and RAMβCD

|  | Diminazene aceturate | HPβCD | Mel/HPβCD | Mel/RAMβCD | Melarsoprol | RAMβCD |
| --- | --- | --- | --- | --- | --- | --- |
| HPβCD | P < 0.0001  (-5.757, -3.346) |  |  |  |  |  |
| Mel/HPβCD | P = 0.0086  (-2.786, -0.375) | P < 0.0001  (1.766, 4.177) |  |  |  |  |
| Mel/RAMβCD | P = 0.0003  (-3.610, -1.198) | P = 0.0007  (0.942, 3.353) | P = 0.2674  (-2.029, 0.382) |  |  |  |
| Melarsoprol | P = 0.0002  (-3.681, -1.270) | P = 0.001  (0.870, 3.281) | P = 0.2002  (-2.101, 0.310) | P = 0.9999  (-1.277, 1.1340) |  |  |
| RAMβCD | P < 0.0001  (-5.757, -3.345) | P = 1.000  (-1.205, 1.206) | P < 0.0001  (-4.176, -1.765) | P = 0.0007  (-3.352, -0.9412) | P = 0.001  (-3.281, -0.8696) |  |
| Mean ± SE  N | 100.6 ± 23.6  3 | 0.00 ± 0.00  3 | 21.62 ± 8.64  3 | 8.80 ± 3.00  3 | 6.983 ± 0.308  3 | 0.00 ± 0.00  3 |

The IC_50_ of each compound was determined against wild type S427 *T. b. brucei* trypanosomes by Alamar blue assay. The figures in the body of the table demonstrate the comparisons, in terms of statistical significance, between the IC_50_ (nM) of each compound, shown in the row and column headings. The p-values and 95% confidence intervals for differences are based on analysis using the logarithmic transformation [log(x+1)] of the IC_50_. The mean IC_50_ value ± the standard error and the number of repeats are also shown.
