# Supplementary material for: Melarsoprol Cyclodextrin Inclusion Complexes as Promising Oral Candidates for the Treatment of Human African Trypanosomiasis
Source: PLoS Negl Trop Dis. 2011 Sep 6;5(9):e1308. doi: 10.1371/journal.pntd.0001308 (PMC3167784; doi:10.1371/journal.pntd.0001308)
Supplement: Table S2 — Copy number of the PFR2 gene detected within the brain following mel/HPβCD chemotherapy. Mice were infected with T. b. brucei GVR35/C1.9. Mel/HPβCD chemotherapy commenced on day 21 post-infection. The compound was administered by orally gavage, daily for 7 consecutive days at dose of 0.05 mmol/kg. The number of copies of the PFR2 gene present within 100 ng of DNA prepared from approximately 25 mg of whole brain homogenate, 24 hours after administration of each dose was determined by Taqman PCR. The figures in the body of the table demonstrate the comparisons, in terms of statistical significance, between the number of copies of the PFR2 gene detected after administration of each dose, shown in the row and column headings. The p-values and 95% confidence intervals for differences are based on analysis using the logarithmic transformation [log(x+1)] of the copy number. The mean copy number ± the standard error and the number of animals per group are also shown. No copies of the PFR2 gene were detected following the 4th treatment or subsequent drug doses these groups have been removed from the analysis. (DOC) [file pntd.0001308.s003.doc]

Table S2: Comparison of the number of copies of the PFR2 gene detected within the brain following mel/HPβCD chemotherapy

| Dose | 1 | 2 | 3 |
| --- | --- | --- | --- |
| 2 | P = 0.0220  (-2.860, -0.218) |  |  |
| 3 | P = 0.0001  (-4.390, -1.748) | P= 0.0228  (-2.851, -0.2088) |  |
| Mean ± SE  n | 68.1 ± 14.7  6 | 13.13 ± 2.54  6 | 4.32 ± 2.51  6 |

Mice were infected with *T. b. brucei* GVR35/C1.9. Mel/HPβCD chemotherapy commenced on day 21 post-infection. The compound was administered by orally gavage, daily for 7 consecutive days at dose of 0.05mmol/kg. The number of copies of the PFR2 gene present within 100ng of DNA prepared from approximately 25mg of whole brain homogenate, 24 hours after administration of each dose was determined by Taqman PCR. The figures in the body of the table demonstrate the comparisons, in terms of statistical significance, between the number of copies of the PFR2 gene detected after administration of each dose, shown in the row and column headings. The p-values and 95% confidence intervals for differences are based on analysis using the logarithmic transformation [log(x + 1)] of the copy number. The mean copy number ± the standard error and the number of animals per group are also shown. No copies of the PFR2 gene were detected following the 4^th^ treatment or subsequent drug doses these groups have been removed from the analysis.
