# Supplementary material for: Melarsoprol Cyclodextrin Inclusion Complexes as Promising Oral Candidates for the Treatment of Human African Trypanosomiasis
Source: PLoS Negl Trop Dis. 2011 Sep 6;5(9):e1308. doi: 10.1371/journal.pntd.0001308 (PMC3167784; doi:10.1371/journal.pntd.0001308)
Supplement: Table S4 — Comparison of the percentage signal change data generated from MRI scans. Mice were infected with T. b. brucei GVR35/C1.9. Immediately prior to treatment commencing on day 21 post-infection, animals were MRI scanned. Following recovery from the MRI procedure animals were administered mel/HPβCD orally at a dose of 0.05 mmol/kg. Mel/HPβCD treatment continued for the next 6 days. Twenty-four hours, 8 and 15 days after administration of the last dose, corresponding to days 28, 35 and 42 post-infection respectively, the MRI scans were repeated. Each MRI scan consisted of 20 continuous coronal slices. The brain was manually selected in each slice and the percentage signal change calculated. The figures in the body of the table demonstrate the comparisons, in terms of statistical significance, between the times post-treatment shown in the row and column headings. The P-values and 95% confidence intervals are based on analysis using the percentage signal change for each slice. The mean signal change ± the standard error and the number of animals per group are also shown. (DOC) [file pntd.0001308.s005.doc]

Table S4: Comparison of the percentage signal change detected generated from MRI scans.

|  | Untreated, uninfected | 21 days post-infection,  Untreated | 24 hours post-treatment | 8 days post-treatment | 15 days post-treatment |
| --- | --- | --- | --- | --- | --- |
| 21 days post-infection, Untreated | *P* < 0.0001  (7.951, 13.576) |  |  |  |  |
| 24 hours post-treatment | *P* = 0.9296  (-1.992, 3.633) | *P* < 0.0001  (-13.02, -6.862) |  |  |  |
| 8 days post-treatment | *P* = 0.2254  (-0.669, 4.956) | *P* < 0.0001  (-11.70, -5.539) | *P* = 0.7622  (-1.758, 4.404) |  |  |
| 15 days post-treatment | *P* = 0.9832  (-3.363, 2.261) | *P* < 0.0001  (-14.40, -8.234) | *P* = 0.7370  (-4.452, 1.709) | *P* = 0.1177  (-5.775, 0.3862) |  |
| Mean ± SE  N | 7.1 ± 0.162  3 | 17.87 ± 1.62  2 | 7.93 ± 0.45  2 | 9.2 ± 0.60  2 | 6.55 ± 0.46  2 |

Mice were infected with *T. b. brucei* GVR35/C1.9. Immediately prior to treatment commencing on day 21 post-infection, animals were MRI scanned. Following recovery from the MRI procedure animals were administered mel/HPβCD orally at a dose of 0.05mmol/kg. Mel/HPβCD treatment continued for the next 6 days. Twenty-four hours, 8 and 15 days after administration of the last dose, corresponding to days 28, 35 and 42 post-infection respectively, the MRI scans were repeated. Each MRI scan consisted of 20 continuous coronal slices. The brain was manually selected in each slice and the percentage signal change calculated. The figures in the body of the table demonstrate the comparisons, in terms of statistical significance, between the times post-treatment shown in the row and column headings. The *P*-values and 95% confidence intervals are based on analysis using the percentage signal change for each slice. The mean signal change ± the standard error and the number of animals per group are also shown.
