# Supplementary material for: Melarsoprol Cyclodextrin Inclusion Complexes as Promising Oral Candidates for the Treatment of Human African Trypanosomiasis
Source: PLoS Negl Trop Dis. 2011 Sep 6;5(9):e1308. doi: 10.1371/journal.pntd.0001308 (PMC3167784; doi:10.1371/journal.pntd.0001308)
Supplement: Table S5 — Parameters defining the injury score allocated to the severity of the neuropathology. Injury sores are given horizontally while the criteria used to define the scores are detailed vertically. (DOC) [file pntd.0001308.s006.doc]

Table S5. Parameters defining the injury score allocated to the severity of the neuropathology.

|  | Score | | | | |
| --- | --- | --- | --- | --- | --- |
|  | 0 | 1 | 2 | 3 | 4 |
| Meningitis | None | Mild | Moderate | Severe | Severe |
| Perivascular cuffing | None | None | Mild cuffing of some vessels | Prominent cuffing of some vessels | Prominent cuffing of most vessels |
| Encephalitis as defined by cellular activity in the neuropil | None | None | None | Moderate | Severe |

Injury sores are given horizontally while the criteria used to define the scores are detailed vertically.
